# Young Spanish People’s Gendered Representations of People Working in STEM. A Qualitative Study

**DOI:** 10.3389/fpsyg.2019.00996

**Published:** 2019-05-07

**Authors:** Milagros Sáinz, José-Luis Martínez-Cantos, María Rodó-de-Zárate, María José Romano, Lidia Arroyo, Sergi Fàbregues

**Affiliations:** ^1^Internet Interdisciplinary Institute, Universitat Oberta de Catalunya, Barcelona, Spain; ^2^Department of Education, Universidad Complutense de Madrid, Madrid, Spain; ^3^Department of Psychology and Education, Universitat Oberta de Catalunya, Barcelona, Spain

**Keywords:** gender stereotypes, role models, portrayals, STEM, under-representation

## Abstract

The present qualitative study analyzes how a group of young people already involved in STEM fields perceive the prototypical person working in STEM. Gender differences between participants in technological and non-technological STEM fields were analyzed. A total of 27 young people (59.3% women) took part in the interviews (Mean Age = 25.48 years). Of them, 16 participants were working in STEM professions, and 11 were enrolled in the final courses of STEM degrees. The results of the content analysis were examined in light of social role theory and the multidimensional structure of gender stereotypes. Men in these fields were therefore attributed an unappealing and weird physical appearance. Some female participants linked STEM professionals’ intellectual abilities to the stereotype that men have higher abilities in these fields. Whereas females attributed effort and perseverance to STEM professionals’ intellectual aptitudes, males referred to the development of soft skills. Participants in technological STEM fields connected the stereotype of being a ‘weirdo’ to a boring job, whereas those in non-technological fields linked it to their unconventional character. Some participants were disappointed by a lack of correspondence between expectations and the actual job STEM professionals do. Moreover, females in technological STEM fields commented on the job’s low social impact, while males mentioned low attainment of technical qualifications. Most referents in STEM fields were masculine, some of whom were present in the mass media. The practical implications of the findings are discussed.

## Introduction

Our society has experienced important advances in terms of equality thanks to the efforts deployed to achieve an egalitarian education among young people. However, we continue observing a marked gender gap in the academic and professional aspirations that young people develop during secondary education (Wang and Degol, 2013; Sáinz and Müller, 2018). In addition, and although they have nearly attained equality with men in several formerly male-dominated fields, women remain underrepresented in several fields of science, technology, engineering and mathematics (STEM), (Wang and Degol, 2013; UNESCO, 2017).

Nowadays, many girls are reluctant to choose STEM disciplines related to engineering, computer science, and physical science. In fact, and according to the Spanish Ministry of Education (MECD, 2018), during the 2016–2017 academic year women represented only 17, 17.39, and 11.83% of the student enrollments in computer science, electrical and energy engineering, and electronics and automation technologies, respectively. However, women are significantly represented in scientific disciplines such as biology, mathematics, and chemistry, accounting for 61.78, 37.66, and 53.20%, respectively, of student matriculation in these university degrees. Above all, women outnumbered men in disciplines related to the provision of healthcare, such as medicine or pharmacy, representing 65.8 and 69.58% of total enrollments in these studies, respectively.

This phenomenon can be also observed in many Western countries. According to data from UNESCO (2017), within the female student population in higher education globally, only around 30% choose STEM studies. As in Spain, differences can be observed by disciplines. Female students’ enrollment is particularly low in information and communication technologies (ICT) (3%), natural sciences, mathematics and statistics (5%), and engineering, manufacturing and construction (8%); but the highest participation is in health and welfare (15%) studies (UNESCO, 2017). These data highlight the importance of analyzing gendered representations of people working in technological STEM fields versus those in non-technological STEM fields. Thus, technological STEM fields include people graduate in areas like engineering, computer science, or architecture. These disciplines are mainly oriented to the design of technological appliances and services and in most of the cases women remain remarkably underrepresented (Sáinz and Müller, 2018). In addition, the non-technological STEM fields group comprises people graduate in science disciplines like biology, pharmacy, medicine, or mathematics, where technologies are frequently the tool rather than the object of their work and women are in general highly represented (Sáinz and Müller, 2018).

### Gender Stereotypes of People Working in STEM

Gender-role stereotyping of careers might be an important reason why women are staying away from many STEM careers (Wang and Degol, 2013; Sáinz et al., 2016a; Steinke, 2017). According to the multidimensional structure of gender stereotypes (Deaux and Lewis, 1984) and Eagly’s (2001) social role theory, gender stereotypes have a multidimensional structure because they comprise features associated with the ideal person working in a particular field (i.e., physical appearance, role behaviors, personality traits, and occupations). People take these characteristics as a reference to make inferences about the ideal man or woman working in different occupations.

According to social role theory, women are thought to behave in a communal fashion—that is, concerned about other people, friendly, and expressive. In contrast, men are thought to behave in an agentic manner—independent, assertive, and instrumental (Eagly, 2001). People in highly male-dominated STEM fields (such as engineering) will therefore be more likely to behave in an agentic way, whereas people in highly female-dominated fields (such as education or nursing) will be more likely to behave in a communal fashion. Thus, people in highly male-dominated STEM fields like engineering will be depicted as having several attributes (such as being weird, possessing high intellectual abilities, developing technical tasks, or earning lots of money) congruent with the masculine agentic gender role rather than with the feminine communal gender role. For the goals and roles congruity theory (Diekman et al., 2010)—a theoretical framework stemming from social role theory—the underrepresentation of women in STEM careers is associated with the perception that STEM careers are less likely than careers in other fields (such as psychology) to fulfill communal goals (e.g., working with or helping other people). Consistent with this theory, these perceptions might disproportionately affect young women’s career decisions in many STEM fields, because women are more likely to endorse communal goals than men (Diekman et al., 2010).

In this regard, research on young people’s portrayal of a typical person working in STEM shows that, when asked to draw a scientist using the Drawing a Scientist Test (DAST) (Chambers, 1983) or describe STEM professionals, adolescents tend most often to depict these professionals as male, as well as unattractive, white, middle-aged or elderly, dressed in a lab coat, wearing glasses, ‘geeky,’ or ‘nerdy,’ socially awkward, and being people who work alone (Barker and Aspray, 2006; Steinke et al., 2007; Cheryan et al., 2013; Sáinz et al., 2016b). Girls were more likely than boys to report the counter gender-stereotyped perception of scientists and STEM professionals as female (Steinke et al., 2007; Sáinz et al., 2016b). In addition, recent studies have corroborated the assumption that women are more likely than men to be underrepresented in many STEM fields because women are stereotyped as being less likely to possess a sort of ‘raw’ talent than men (Meyer et al., 2015). Most of these portrayals are related to people working in scientific or technological fields, such as physical scientists or engineers (Steinke et al., 2007; Sáinz et al., 2016b). Moreover, several studies show that male-dominated jobs such as engineering and other technology-related occupations are associated with a high status and well-paying stereotype (Eagly, 2001; Sáinz et al., 2016b). That is, young people’s portrayals of STEM professionals include different features that make reference to the person’s physical appearance and other several gender role behaviors (Eagly, 2001; Sáinz et al., 2016b). These portrayals are important to examine because they shape young people’s interest in pursuing STEM courses and occupations (Steinke et al., 2007). For this reason, in the present research we aim to study the gendered representations that a group of young people (some of them already in STEM) have about a typical person working in various STEM fields beyond engineering and physical science. Given the disparity of women’s representation across STEM fields, differences in gender and discipline will also be analyzed.

Schools, families, and popular media such as TV series and Hollywood movies play a crucial role in the construction, representation, reproduction, and transmission of stereotypes of these STEM professionals (Steinke, 2017). In this regard, gender stereotypes of STEM professionals in the media influence students’ stereotyped perceptions of STEM (Steinke et al., 2007; Steinke, 2017). This information is particularly salient and relevant for girls and boys during and beyond adolescence, as young people actively consider their future personal and professional identities not only before selecting any concrete field, but also after having selected it and deciding to develop professionally in that area. Incidentally, it can be expected that the stronger the correspondence between young people’s self-portrait and the archetypal person working in a given field—for instance a science teacher—the more likely the young person is to choose this field (Kessels and Taconis, 2012).

### Gender -Marking Language to Express Stereotypical Portrayals of People in STEM

The use of masculine, feminine, and neutral gender marks provides researchers with interesting additional information about the way young people depict the typical image of someone working in the different STEM fields (Gabriel and Gygax, 2016). As demonstrated in other studies, the use of the masculine generic in Indo-European-origin languages with grammatical gender, such as Spanish, Catalan, and French, denotes a high underrepresentation and undervaluing of women in many STEM fields (Gabriel and Gygax, 2016; Sáinz et al., 2016b). The existence of semantic gender markers in Spanish and Catalan (languages used in the context of the present research) activates gender categories and the perpetuation of differing expectations for men and women. It also reinforces existing gender stereotypes (Gabriel and Gygax, 2016). For instance, the use of the feminine singular enfermera or infermera to refer to a female nurse in Spanish and Catalan, or the usage of the masculine singular to refer to a doctor in both languages as médico or metge, or the generic plural masculine to refer to different professions such as engineers, physical scientists, or scientists either in Spanish—ingenieros, físicos, científicos— or in Catalan—enginyers, físics, científics—. These gender markers are not only limited to nouns, but also apply to pronouns and adjectives. In a recent research study conducted in the context of Spain, secondary students associated more masculine than non-masculine references to a person working in a highly male-dominated field such as information and communication technologies (Sáinz et al., 2016b). These workers were associated with masculine characteristics through the use of adjectives and other markers.

### The Present Study

There is a lack of research focused on gendered portrayals of people working in STEM fields with high numbers of women, mainly with a non-purely technological orientation such as biology, biomedicine, or chemistry. In the present research we thereby simultaneously examine the opinion that a group of participants belonging to highly male-dominated STEM fields (with a high technological component, such as engineering or computer science) and highly female-dominated STEM fields (with a less technological orientation) have about the typical person working in the STEM field. Similarly, there is no research about the image that young people already in STEM hold regarding the typical person working in STEM. In this regard, most research looking at young Spanish people’s portrayals of professionals has been conducted with secondary students (Sáinz et al., 2016a,b). In addition, most of the research on these aspects has been conducted via surveys and using various mixed methods. Qualitative research delving into the type of stereotypical gender role portrayals of people already in STEM fields is scarce. For this reason, the present study applies a novel qualitative approach to examine young people’s gendered representations of people working or studying in different STEM fields. Through this research, we therefore attempt to cover the aforementioned research gaps.

The research questions and hypotheses were therefore formulated as follows:

(RQ1) What are the main features that participants highlight as portraying the typical person working in—technologically and non-technologically oriented—STEM fields?
H1: Participants are expected to report more masculine than feminine characteristics (i.e., physical appearance, intellectual abilities, personality traits, or social position) when describing the prototypical person working in technologically, and non-technologically–oriented STEM fields. (RQ2) To what extent do male participants differ from female participants in their portrayals of the prototypical person working in STEM?
H2: More male than female participants are expected to provide masculine features when portraying the prototypical person working in STEM.
(RQ3) To what extent do participants studying or working in technological STEM fields express their portrayal of the prototypical person working in STEM in the same terms as participants in non-technological STEM fields?
H3: More participants in technological STEM fields than in non-technological STEM fields are expected to use masculine features when describing the prototypical person working in the field.
(RQ4) To what extent do male and female participants from technological STEM fields express their portrayal of the ideal of the person working in STEM in similar terms as male and female participants from non-technological STEM fields?
H4: In comparison to male participants in non-technological STEM fields, male participants in technological STEM fields will use more masculine features to portray the person working in STEM. The same would be true for female participants in technological STEM fields, in comparison to female participants in non-technological STEM fields.

## Materials and Methods

### Study Design

A qualitative descriptive design (Sandelowski, 2010) was used to generate an accurate and in-depth account of how young people already in STEM perceive the prototypical person working in this field. This type of design is especially suited to research situations where researchers want to use a low level of interpretation of the events studied. In contrast to more interpretative qualitative approaches such as grounded theory, phenomenology, or ethnography, in which “a conceptual or otherwise highly abstract rendering of the data” (Sandelowski, 2010, p. 335) is required, in qualitative descriptive studies researchers stay close to the data by presenting the facts in the everyday language of the participants. Therefore, this approach enabled us to ensure descriptive validity (Maxwell, 1992), that is, to gain an accurate understanding of participants’ thoughts and beliefs, expressed in their own words and, as a result, minimize researcher bias. Furthermore, the adoption of a qualitative descriptive approach was consistent with the primary goal of describing and understanding the subjective nature of the perceptions conveyed by the participants.

### Sample

Purposive sampling was used to select 11 students in the second or higher year of their bachelor’s degree and 16 STEM professionals employed in the private sector for 1–5 years.

We aimed for heterogeneity in both groups in terms of gender and type of degree program. Potential participants were identified using formal and informal strategies, including the following: (a) Asking acquaintances if they knew of any potential participants; (b) contacting student associations, professors, and companies in the STEM field; and (c) snowballing from previous contacts. We continued to interview until data saturation was achieved, that is, new data generated no further insights. Saturation was assessed by analyzing the interview transcripts. Consequently, 27 participants were included in the study. The sample size was consistent with recommendations suggested in the literature (Kuzel, 1999; Guest et al., 2006). Before being interviewed, participants were individually screened by telephone or email to ensure eligibility criteria were met.

The characteristics of the 27 study participants are displayed in Table 1. Participants included 11 males and 16 females, either finishing the last course of a STEM university degree (five males and six females) or working in a STEM field with a maximum of 5 years’ experience in private companies (6 males and 10 females). Participants were living in the metropolitan areas of Barcelona (11 students and 8 professionals) and Madrid (8 professionals). The mean age of participants was 22.6 (*SD* = 1.4) for students and 27.4 (*SD* = 2.9) for professionals. The students were enrolled on degree courses in physical sciences (*n* = 3), computer science engineering (*n* = 2), telecommunications engineering (*n* = 2), mathematics (*n* = 1), medicine (*n* = 1), pharmacy (*n* = 1), and physics engineering (*n* = 1), whereas the professionals had completed degrees in industrial engineering (*n* = 3), architecture (*n* = 2), biology (*n* = 2), pharmacy (*n* = 2), physical sciences (*n* = 2), telecommunications engineering (*n* = 2), aeronautical engineering (*n* = 1), mathematics (*n* = 1), and mining engineering (*n* = 1). All the participants were born in Spain.

**Table 1:** Characteristics of the 27 study participants.

	Student (*n* = 11)	Professional (*n* = 16)	Total (*n* = 27)
Mean age (*SD*)	22.64 (1.50)	27.44 (2.99)	25.48 (3.43)
Gender, *n* (%)			
Male	5 (45.5)	6 (37.5)	11 (40.7)
Female	6 (54.5)	10 (62.5)	16 (59.3)
Place of residence, *n* (%)			
Barcelona	11 (100)	8 (50)	19 (70.4)
Madrid	0 (0)	8 (50)	8 (29.6)
STEM field, *n* (%)			
Technological	5 (45.5)	9 (56.3)	14 (51.9)
Non-technological	6 (54.5)	7 (43.8)	13 (48.1)
Bachelor’s degree, *n* (%)			
Technological STEM fields			
Aeronautical Engineering	0 (0)	1 (6.25)	1 (3.7)
Architecture	0 (0)	2 (12.5)	2 (7.4)
Telecommunications Engineering	2 (18.2)	2 (12.5)	4 (14.8)
Computer Science Engineering	2 (18.2)	0	2 (7.4)
Industrial Engineering	0 (0)	3 (18.75)	3 (11.1)
Physics Engineering	1 (9.1)	0	1 (3.7)
Mining Engineering	0 (0)	1 (6.25)	1 (3.7)
Non-technological STEM fields			
Medicine	1 (9.1)	0	1 (3.7)
Pharmacy	1 (9.1)	2 (12.5)	3 (11.1)
Physical Sciences	3 (27.3)	2 (12.5)	5 (18.5)
Mathematics	1 (9.1)	1 (6.25)	2 (7.4)
Biology	0 (0)	2 (12.5)	2 (7.4)
Mean years since degree completion (*SD*)	–	4.06 (2.35)	–
Mean years of work experience (*SD*)	–	3.94 (1.48)	–


### Data Collection

Semi-structured interviews were conducted from April to September 2016. All interviews took place in Spanish in locations chosen by the participants, such as campuses, workplaces, and coffee shops. The interviews lasted from 40 to 90 min and were conducted by four members of the research team. The majority of the interviews were conducted in Spanish, but six of them were held in Catalan. Follow-up prompts were used to allow interviewees to expand on their answers. Before the interviews, we obtained informed consent and authorization to record the responses.

The interview guide, based on the research questions and a review of the literature, included the following three questions: (1) Why did the participants decide on a STEM degree? (2) How would they characterize a prototypical STEM professional? (3) What do they consider to be the significant barriers to and facilitators of women’s access to the STEM field? Each question had the same weight and allotted time in the interview. However, only the findings related to the second question are reported and discussed in this article.

### Data Analysis

Interviews were transcribed verbatim, imported into QSR NVivo software, and analyzed using qualitative content analysis (Schreier, 2012). This method allowed us to focus on categories of interest in the interview data set and systematically analyze them in a flexible way. Qualitative content analysis was implemented in three phases. First, the coding scheme was drafted based on Deaux and Lewis (1984) theory of the multidimensional structure of gender stereotypes. The coding scheme included various codes, including the following: intellectual aptitudes, personality traits, social position, and role models associated with the various STEM disciplines. In addition, we added the code of Spanish gender-marked terminology referring to professionals in these disciplines (e.g., *enfermera*, *médico*, *ingeniero*, and *arquitecto*). Second, to test the coding scheme, two researchers applied it to the same 30% of the data using NVivo. Results were compared and the researchers discussed those cases in which the same segments of text were assigned different codes. The coding comparison ensured that the two coders interpreted the codes similarly and facilitated evaluation of the consistency and validity of the coding scheme. Disagreements were discussed and arbitrated by a third member, when necessary. A few changes were made as a result of this test. These included merging similar codes and eliminating those that were found to be irrelevant. In the third phase, we applied the coding scheme to the interviews. After all the data were coded, NVivo matrix coding query was performed in order to compare responses with the characteristics of the interviewees (i.e., gender, STEM field, bachelor’s degree).

## Results

### Physical Appearance

Several instances regarding the physical appearance of people working in the different STEM fields were identified. However, whereas the prototypical image associated with most people in these STEM fields had a positive formal look (with descriptions such as ‘a person with glasses and wearing a white coat’), computer and physical scientists were mainly associated by some participants with a ‘weird’ and sometimes negative unattractive physical image (‘untidy,’ ‘careless,’ ‘with uncombed hair,’ or ‘pale skin’). Consistently, most of these prototypical people were explicitly associated with men. The use of gender marks (masculine nouns, attributes, or complements) was evidence of this masculine portrayal. The next description of a male computer science student exemplifies that masculine portrayal.

With dark clothes, a bit heavy metal-looking (masculine). A bit pale (masculine), spending all the time confined to a room under a florescent light without daylight, […] the typical freak spending hours on the computer with a bag of Cheetos by his side. (Participant 1)

No differences were observed among the participants with technological and non-technological STEM backgrounds. In addition, both male and female participants expressed similar stereotypes about the physical appearance of STEM professionals. However, a gender bias emerged since the stereotype about the unkempt appearance of professionals in highly male-dominated fields was exclusively related to masculinity. In general, both male and female participants considered that women take much more care of their physical appearance, which according to them could discourage women from entering fields where physical appearance is not important or which involve dealing with raw materials and wearing coveralls. The following testimony of a female pharmacist working in a lab refers to those aspects.

In the production department everything was very dirty, you handle lots of materials, raw material […] it’s more for boys. (Participant 2)

In this regard, for a female engineer the notion that only ‘intelligent and ugly women enter these masculine fields’ discouraged many young girls from entering these professions. She also explained how at university she changed her physical appearance (abandoning the use of make-up and high heels) in order to adapt to the way her female engineering university colleagues dressed and looked.

I entered university wearing high heels. I used to wear make-up, but none of my classmates did. Then I started to wear low-heeled shoes, dress more casually, comfortably, […] engineering […] is something very *macho*, it’s like people considered that only the ugliest and brightest women entered the field […], which could be a reason for women not studying engineering, you get your hands dirty. (Participant 3)

In fact, a couple of female scientists believed that they did not fit into the stereotype of someone who does not take care of their physical appearance. In general terms, the results are in line with previous studies (Cheryan et al., 2013; Sáinz et al., 2016b) and show participants’ use of features regarding the physical appearance of STEM professionals when referring to their prototypical image.

#### Intellectual Aptitudes

In general, different attributes associated with intelligence were reported by many participants when describing the prototypical person working in STEM fields. No differences between participants from technological and non-technological STEM fields were identified. However, differences between male and female participants were observed. On the one hand, male participants (like the engineer quoted below) linked intelligence to technical, spatial, mathematical, and/or physical science abilities.

I imagined a man with good mathematical or physical science abilities, with a lot of technical knowledge and good spatial abilities. (Participant 4)

Some male participants with work experience (like the next engineer) also underlined the need to possess certain soft-skills (being open-minded or having good managerial and business skills) or good personal qualities (especially if working with clients) as a complement to technical skills.

Everyday good managers are in demand. It is not only a matter of being technically qualified. You also have to understand the fiscal and economic implications of your work. (Participant 5)

On the other hand, some female participants (such as the following telecommunications engineering student) considered that hard work, perseverance, and effort were basic dimensions of the intelligence associated with STEM professionals.

It is obvious that having the ability is essential, but effort is also important. (Participant 6)

Likewise, some female engineers remarked that having high intellectual abilities did not mean being educated; it could be connected with STEM professionals’ lack of social skills. The next female telecommunications engineer suggests a lack of cultural knowledge among engineers, despite their high intellectual abilities.

They are intelligent people, who know to compute a partial derivative in 20 s, but maybe they don’t know what the capital city of Kuwait is. (Participant 7)

Moreover, some female participants like the following graduate in biomedicine talked about further aspects of intelligence related to STEM professionals’ personality traits (e.g., being methodical, capable of resolving problems, rigorous, or highly creative). That is, people with flexible intellectual aptitudes, strong analytical skills, and logical reasoning.

With an analytical vision—not narrow-minded, but analytical, objective—of how to plan things with sound logical reasoning; but this does not exclude a more intuitive side. (Participant 8)

Finally, it is important to note that a couple of female participants highlighted a relationship between the stereotype of STEM professionals’ high intellectual abilities and sexism in the field. For them, intelligence tends mainly to be considered a masculine characteristic. Another female engineer commented on the common assumption that women have less technological abilities than men, and for this reason, women were supposed to stand out because of their good communication and organizational skills.

I don’t know why it is supposed that women have less knowledge about technologies. In my field, when you are doing an interview, unconsciously, they think that you have fewer abilities. Maybe they expect you to make up for that gap in your technological abilities with other qualities such as being more organized, getting on well with other people, having more fluid communication skills. (Participant 7)

A female interviewee in the life sciences explained that she had held the prejudice that men were the best and most outstanding scientists.

At the research level, I had the mindset that women also did research, but I always believed a certain […] cliché that men were better. (Participant 9)

As observed, and in line with the theoretical background (Deaux and Lewis, 1984; Eagly, 2001), many students referred to several aspects of the intellectual aptitudes associated with people working in STEM.

#### Personality Traits

Participants alluded to aspects related to STEM professionals’ character or personality traits. No gender differences emerged regarding their view of STEM professionals’ personality traits. In this sense, both male and female participants belonging to technological STEM settings (a total of 14 interviewees) characterized professionals in these fields in terms of being ‘freaks’ or ‘weirdos.’ In the same fashion, the term *freak* was used by some participants in the fields of math and physical science to describe people working in these fields. However, both male and female participants mainly referred to engineers, computer scientists, or physical scientists as people (normally men) lacking communication skills (i.e., a grumpy male, confined to his room, or a person lacking empathy). Remarkably, one male participant even alluded to physical scientists as male heterosexuals.

Moreover, computer and physical scientists were described by participants from both genders as males with a clear focus on activities that could be boring for other people (i.e., obsessed, lunatic, or a ‘bookworm’). Interestingly, many of these participants (like the following male computer engineering student) also referred to STEM professionals as lacking team-building abilities.

A bookworm, a grumpy male, a person confined to his/her room. As the machine does not allow human interaction […]. Little empathy. (Participant 10)

In addition, whereas participants in technological STEM fields (like the male aeronautical engineer referred in the next first quote) described those working in these fields as people who found it difficult to establish social relations (‘grumpy’) or had an analytical mind or little empathy, participants in non-technological STEM fields (like the male physical science student mentioned in the following second quote) portrayed people working in scientific STEM fields as ‘lively,’ ‘amusing,’ ‘spontaneous,’ ‘extroverted,’ and also ‘weird,’ but in terms of being independent and unconventional.

Yes, with difficult personal relations. (…) like Sheldon Cooper, who thinks that his work is more important than what others do; a man or woman who is passionate about what he/she is studying or working on (…) obsessed about this. (Participant 11)

I imagined physical scientists a bit like mathematicians, stereotypically more spontaneous. They do not follow social conventions. (Participant 12)

Remarkably, in comparison to the two participants from the field of architecture (who portrayed architects as being bohemian or artists), a participant in the field of engineering portrayed engineers as being serious, entrepreneurial, and practical because they were supposed to get straight to the point. On the other hand, participants in STEM health-related fields (mostly women such as the pharmacy student mentioned in the next quote) commented on these STEM professionals’ kindness and predisposition to help people.

A very serious person, […], kind. A person who can give you a hand, an honest male, very upright. (Participant 13)

Likewise, and aligned with their vision of the intellectual aptitudes associated with STEM professionals, female participants (like the biomedicine worker cited in the following quote) placed greater emphasis on personality traits related to dedication, perseverance, and seriousness. Conversely, male participants were more focused on describing STEM professionals as independent people who do not follow social conventions.

A well-considered person, a hard-working person, with intellectual capacity, a serious male. Strict and dogmatic people. Willing to work many hours without being paid. (Participant 9)

Moreover, most male and female participants believed that the stereotype regarding STEM professionals’ lack of communication skills fitted more with male rather than female examples, given that women were supposed to be more communicative and empathetic. However, some female participants complained about how the stereotype of women’s poor technical competences led them to assume tasks congruent with this stereotype. That is, to join teams to develop social and communication skills rather than technical skills. In the following testimony, a male engineering student attempts to dismantle the stereotype that women have more communicative skills than men.

Women, […] empathize more easily given the work that they have unconsciously achieved, but […] I have also seen disastrous presentations given by girls; girls who do not know how to communicate. (Participant 14)

Some participants (as illustrated in the testimony of the next male physical science student) also acknowledged that the image they had of a person working in the field had changed after having entered into contact with real people either at university or work.

Physical scientists are not actually like I imagined; they are a bit crazy and extroverted, but a bit serious and in this regard a bit different from what I expected. (Participant 1)

All these testimonies inform us about the importance of considering personality traits when tackling the portrayal of a typical person working in STEM.

#### Social Position

Explicit reference was made to aspects related to the status or social position of people working in STEM fields. In this regard, some male and female participants stated that STEM jobs were generally well considered because of the associated prestige (or social importance, as represented in the own words of a male architect in the next first quote), salary, and respect (these two aspects are commented by a male engineer in the following second quote), or the content of the tasks to be developed. Equally, there is the belief that people do not fully understand the type of work carried out by many of these STEM professionals.

With high social importance. (Participant 15)

It is well-paid […] you suffer for some years, but then people outside began to respect me. (Participant 3)

However, the two male architects participating in the study complained about the low salary and lack of stability associated with jobs in the field, mainly in comparison to the years prior to the economic crash (that took place in Spain between years 2008 and 2011 with negative effects particularly on the real-state sector) and to other participants in the fields of engineering and computer science who did not mention any of these aspects.

My salary is basically my main barrier because I consider that with my background I should have a higher salary. I have a technical degree, architecture, I speak four languages, I have an international career, I have done international projects and I believe that people in Spain do not value this. (Participant 16)

On the other hand, women in non-technological fields (mainly in health) felt that their jobs were very well considered, as long as they were associated with respect and admiration. Nevertheless, in comparison to technological STEM fields, participants in biology-related jobs like the next woman complained of the low pay and poor labor conditions, in spite of being well considered socially.

On a labor level, a low-paid person. […] Socially speaking, quite the opposite, that is, a well-considered person, in terms of being hard-working, and with intellectual capabilities. (Participant 9)

Some participants also mentioned that a pharmacist’s (male) status was lower than that of a medical doctor (male). Likewise as defended by the following female medicine student, a medical doctor (male) had more ‘knowledge’ than a nurse (female). Interestingly, the singular masculine was associated with a person working as a medical doctor (*médico* or *metge*) or a pharmacist (*farmacéutico* or *farmacèutic*), whereas the feminine singular was used to refer to a person working as nurse (*enfermera* or *infermera*).

The knowledge acquired by a medical doctor (masculine) is greater than that of a female nurse […] The female nurse has a lot of contact with the patient […], but for making decisions and knowing why things happen, there are things that the (male) medical doctor knows that the (female) nurse has not studied. (Participant 17)

Finally, some participants in the field of health also mentioned a change in the current condition of the pharmacy profession, given that in the past it used to be associated with ‘very wealthy’ people, who had the resources to open a pharmacy. The next female pharmacy student also commented that medical doctors have a higher social consideration than pharmacists.

I cannot see differences between a medical doctor and a pharmacist (both in masculine), but the medical doctor has higher status than the pharmacist. (Participant 13)

In general terms, the social standing of people working in the different STEM professions was part of the stereotypical view of the different STEM fields.

#### Type of Tasks STEM Professionals Do

This category refers to participants’ views of the tasks carried out by people working in the different STEM fields. Most participants in the fields of engineering and physical science believed that, at the time of choosing their university degree, they did not know much about the actual activities and tasks performed by professionals working in each particular field. In fact, for those with work experience, the actual job had little to do with the previous image they had had. In this regard, some of these participants (like the male aeronautical engineer referred in the next quote) reported that their expectations revolved around a professional more dedicated to the manufacturing process (including aspects such as design, calculus, or analysis), and less involved in the performance of managerial or business-related tasks.

In 2009 or 2010 the view that I had of engineering was related to design, manufacturing, calculus, analysis, and maintenance […]. I thought of the tasks that I like to do as an engineer […]. But I know that aeronautical engineering is more than that. (Participant 11)

On the other hand, few male participants such as the following mining engineer admitted to having an erroneous previous image of the tasks carried out by engineering professionals, given that they had an idealization of these professionals.

Sometimes when I say that I am a mining engineer, people associate me with being underground, in the dark, breathing dust. (Participant 18)

Interestingly, two engineering students (one male and one female) reported being deeply disappointed with the gap between the expectations they had about the concrete tasks carried out in each STEM field and what they actually experienced at university. In this regard, a gender difference also emerged, since the female student felt she had been ‘deceived’ because of the lack of social impact of the professional activity (i.e., doing something that changes humanity).

I imagined some genuine work, to investigate something that changes humanity, but then you say: ‘but this, they’ve conned me.’ (Participant 6)

The male student was disappointed because he expected to have attained more technical knowledge (e.g., understanding computer-related processes).

I got upset […] I used to believe that computer science was […] people who knew how to handle a computer, understood it and could work with it. (Participant 18)

However, some female participants in health occupations like the following pharmacy student also indicated a certain degree of knowledge about the tasks to be performed in these occupations.

I imagined what I wanted to become, what I have done in my master’s degree, that is, developing new products in different settings, chemistry, cosmetics, food. (Participant 2)

In line with expectations, most participants referred to the specificities of the tasks that are performed in the different STEM fields when thinking about someone working in the field.

#### Role Models

This category involves the concrete references (significant people like family members, secondary teachers, neighbors, or characters in TV series or movies) that participants mentioned with regard to people working in STEM fields. Both women and men agreed on the idea that people working in the fields of engineering and physical science were predominantly men. The historically higher visibility of men in scientific and technological fields in the media or other public spaces has contributed to this underrepresentation of women. However, some female references such as participants’ mothers or female teachers were mentioned by the interviewees from life science disciplines such as biology, pharmacy, or medicine. Moreover, female referents were thought to take care of their physical appearance, as well as being more responsible, empathetic, predisposed to help others, and with more social skills than men. Male referents, however, were associated with high intellectual and research capabilities, along with greater physical force, and roughness.

Similarly, most male participants in the fields of engineering, architecture, and physical science more frequently spoke about pre-eminent male role models such as Stephen Hawking, Albert Einstein, or Richard Feynman in the field of physical science. Equally, in the field of computer science, several outstanding male figures (such as Mark Zuckerberg or Edward Snowden) were mentioned, as well as some characters in TV series and movies (such as *Mr. Robot*, or Sheldon Cooper from *The Big Bang Theory*), cartoons, or science books. The following testimony of a male computer engineering student revolved around those famous media characters.

When I was I child I used to watch cartoons where inventions, inventors making machines appeared […] I admired the creator of Facebook […] I also paid attention to Snowden […] Right now I am following a TV series about people in Silicon Valley […] *Halt and Catch Fire* […] *Mr. Robot* […] is about security and hackers. (Participant 1)

But also they referred to close male family members and role models such as uncles, parents, teachers, university professors, company leaders, etc. The next quote of one male engineer illustrates this idea.

I had a female professor teaching on engines, she taught very well […] one of the managers in my company […] he works very well. (Participant 3)

Similarly, among most female engineers and physical scientists, male role models also predominated. However, and in line with the next female engineer’s statement, these male figures were the participants’ fathers or secondary school teachers.

My father had an electronics and telecommunications company […] since I was a child […] I wanted to become like my father when I grew up. (Participant 19)

Interestingly, female interviewees rarely mentioned famous figures as their role models. Only one engineer referred to Leonardo Da Vinci as a male role model and to Marie Curie as a female role model. In addition, one male architect complained about having not had any idea during his university training about Zaha Hadid, one of the most inspiring modern architects.

Zaha Hadid, the most famous female architect in the last years, who died recently […] I did not know anything about her, practically until I left university. Participant 13)

In this regard, only a few female engineers mentioned close female role models (such as cousins) working in STEM fields who had inspired them. The next female telecommunications engineer talked about her cousin as her closest female role model.

It was a female cousin […]. When I was a child everybody said ‘Ah, Maria Jesús is doing an engineering degree, it is very difficult […]’ Everybody admired her, […] I somehow aspired to be like her. (Participant 3)

Furthermore, and unsurprisingly, female participants in life science disciplines predominantly identified female referents (i.e., relatives, friends, or teachers) as their role models or mentors. They did not mention experts or characters present in the media.

## Discussion

In general terms and congruent with the multidimensional structure of gender stereotypes (Deaux and Lewis, 1984; Eagly, 2001), the results of the present study confirm the co-existence of various stereotypical attributes about people working in different STEM fields, both technologically and non-technologically oriented. These features reinforce traditional masculine views of people working in STEM, but they also reflect current changing roles of women in the STEM workplace and our society. Some of these stereotypical portrayals are very much associated with gender roles more or less congruent with STEM fields (Diekman et al., 2010). In this regard and in line with expectations (Cheryan et al., 2013; Sáinz et al., 2016b), several marked gender stereotypical portrayals of people working in male-dominated STEM fields such as engineering and computer science were observed. However, in light with expectations more male than female participants provided masculine features when portraying the prototypical person working in STEM. In addition, some intergroup differences with regard to participants’ gender and belonging to technological and non-technological STEM fields have been identified. This is one of the novel contributions of the present research.

As regards the features selected to describe professionals belonging to different STEM occupations, the association of many of these professionals with their physical appearance is evident. These characteristics are aligned with other studies conducted in light of social role theory (Cheryan et al., 2013; Sáinz et al., 2016b). Consistent with expectations (Steinke et al., 2007; Cheryan et al., 2013; Sáinz et al., 2016b), both male and female participants agreed on labeling the stereotypical image of people working in STEM through the use of masculine physical features typically associated with these professionals. In some STEM professions (e.g., physical science or computer science) these physical features were negative and unattractive (uncombed hair, careless, ugly, working in the dark) and opposed to the typical portrayal associated with women, who according to some participants are more likely to take care of their physical appearance. Interestingly, no differences were observed in how participants from technological and non-technological STEM occupations referred to masculine attributes associated with the physical appearance of people working in STEM. For some female participants in technological STEM fields, the lack of possibilities for women to develop their feminine identity in highly masculine STEM occupations such as engineering may discourage them from entering these fields.

In addition and congruent with other studies (Cheryan et al., 2013; Meyer et al., 2015; Sáinz et al., 2016a), attributes related to the possession of intellectual abilities were a major feature mentioned by many participants. No differences between people from technological and non-technological fields emerged. However, for some female participants, these intellectual abilities were associated with other skills related to personality traits that revolve around effort, hard work, or perseverance. But for some male participants, such intellectual abilities were mainly associated with technical capabilities (e.g., mathematical or spatial thinking) or the development of complementary soft skills (e.g., good managerial skills). These findings confirm the hypothesis that STEM fields are above all associated with a high level of raw intellectual capabilities (Meyer et al., 2015). This could also be interpreted as a recognition of communal goals taking an important role in the development of intellectual skills across the different STEM fields (Eagly, 2001; Diekman et al., 2010).

In relation to STEM professionals’ personality traits and in agreement with predictions (Cheryan et al., 2013; Sáinz et al., 2016b), some negative characteristics (such as being antisocial, ‘freaks,’ and only interested in machines) were attributed mainly to people working as scientists, engineers, or computer scientists; that is, highly male-dominated occupations. However, with the exception of physical scientists and mathematicians, other non-technological STEM professionals (that is, highly female-dominated occupations) were less likely to be associated with weird personality characteristics. In line with expectations, more male than female participants provided more masculine features related to personality traits when portraying the prototypical person working in STEM. In addition, being a ‘weirdo’ or a ‘freak’ meant something different for professionals belonging to technological and non-technological STEM fields. Whereas ‘being a weirdo’ in non-technological STEM fields was identified with having a peculiar, independent character, in technological STEM fields it was identified with lacking social skills and performing boring tasks. This result illustrates how according to predictions, more participants in technological STEM fields referred to masculine features when depicting the prototypical person working in the field. Interestingly, whereas male participants in physical science and engineering fully identified with the prototypical negative weird image of these professionals, female participants in these male-dominated STEM fields did not identify with it. In agreement with empirical research (Cheryan et al., 2013; Steinke, 2017), this was considered by some female participants as a factor that detracts many girls from entering in these fields. These findings also suggest that in comparison to male participants in non-technological STEM fields, male participants in technological STEM fields use more masculine features related to physical appearance when portraying the person working in STEM. The same is true for female participants in technological STEM fields, in comparison to female participants in non-technological STEM fields.

Participants from different STEM fields highlighted some stereotypical requirements demanded by the specific field they belong to. For instance, possessing a bohemian character was associated with architecture, whilst being a practical and serious person was part of engineering’s portrayal. Moreover, health professionals were linked to a high predisposition to help others. Interestingly, and with regard to gender differences, some female participants in male-dominated STEM fields such as engineering assumed the stereotype that women are more socially, but less technically, skillful. In addition and in line with predictions, for many female participants, the stereotype regarding STEM professionals’ lack of social skills fitted more with males than with females (Cheryan et al., 2013; Sáinz et al., 2016b). For this reason, female participants did not identify with the stereotypical portrayal of STEM professionals as lacking social skills. In fact, female participants tended to highlight the link between intelligence and personality traits, such as perseverance, dedication, or seriousness. Interestingly, women already in STEM contradict the stereotype regarding people in this field as possessing a high level of raw intellectual ability (Meyer et al., 2015). These women highlight the need to possess complementary attitudes, such as effort or interest in developing the supposed technical intellectual mindset.

With regard to the prestige of STEM occupations, most of the participants coincided in highlighting that STEM degrees were prestigious because of the highly competitive entry prerequisites, difficulty, the social contribution they make, or the high salary associated with some engineering programs (Sáinz et al., 2016b). In harmony with expectations, many of these prestige-related features are congruent with the attainment of masculine agentic goals (Eagly, 2001; Diekman et al., 2010; Sáinz et al., 2016b). However, interesting differences were observed across the various STEM fields. Some architects and biologists complained that these occupations were significant in terms of social recognition, but lacked a decent salary and job stability. Within the health professions, different reputational features emerged, for instance between a medical doctor (in masculine terms) and a nurse (in feminine terms), or a medical doctor (in masculine terms) and a pharmacist (in masculine terms). These findings suggest a certain devaluation of STEM occupations with a high presence of women.

In reference to the type of tasks STEM professionals perform, many participants complained about the lack of information they had when they were in secondary school, at a time when they were deciding on their future studies, regarding the specific work that STEM professionals carry out. Similarly, all the participants with work experience (regardless of the STEM discipline) stressed their disillusionment with the lack of correspondence between the expectations they had regarding the work they would be doing and the actual job. However, whereas male participants in technological STEM fields complained about their disillusionment with regard to the development of technical tasks, female participants in these technical fields complained about their disenchantment in terms of social impact. This last aspect confirms the adherence of some participants’ expectations and professional goals to existing gender roles in highly male-dominated fields (Eagly, 2001; Diekman et al., 2010). That is, whereas women expressed high expectations of attaining communal goals through STEM occupations with a high technical orientation, men expressed high expectations of attaining agentic goals through these STEM fields. These findings also confirm how in comparison to male participants in non-technological STEM fields, male participants in technological STEM fields use more references to the attainment of masculine agentic goals when portraying the typical person working in STEM.

Finally, and congruent with predictions and other research findings (Cheryan et al., 2013; Sáinz et al., 2016a,b), most role models in STEM fields were mainly masculine (i.e., close family members, university professors, secondary teachers, and workmates), particularly in male-dominated fields such as physical science, computer science, or engineering (Steinke, 2017). However, participants in life science occupations mentioned that, although the presence of women in these professions was considerably high, men’s contribution to the field was more salient and men therefore had a better reputation than women in these occupations. Furthermore, while some male participants from technical STEM fields and physical science underlined that some male characters in the media, along with male scientists and science writers, had inspired them, female participants made no such mention. This is further evidence of the lack of female STEM role models in the media. In fact, some participants pointed out how the contribution of women to some STEM fields was not made visible in different STEM fields’ education programs.

### Contributions

The present study contributes to the literature on young people’s portrayals of several STEM fields beyond computer science and technological fields. It is a novel attempt to bring disciplines with a high presence of women, such as medicine and other life science occupations, into the loop of what must be considered as a STEM field. Most studies about these issues do not refer to biological sciences in STEM nor incorporate the prototypical ideal of someone working in biological sciences (Sáinz, 2017). In addition, it provides empirical evidence on how young people already in STEM university studies and jobs perceive the typical person working in STEM. The participants in this research had the opportunity to see the extent to which the stereotypes they had about people working in STEM before entering the STEM field as students or workers had been accurate.

### Limitations and Future Research

This study has four relevant limitations. First, the study used a small sample and, therefore, the findings may not be representative of the views of all the STEM students and young professionals residing in Spain. Variations in the educational and cultural backgrounds of the participants may lead to different representations of people working in STEM. Future cross-cultural research could be conducted with a larger sample of STEM students and workers that incorporate their ethnical and educational backgrounds. Second, the precise prevalence of theme endorsement by the interviewees was not possible to determine. The representations expressed by the participants were identified from interview questions and not from a predetermined list of representations. Consequently, the fact that a representation was not mentioned by a participant does not mean that this representation was not endorsed by the participant. Forthcoming international research could incorporate a systematic way of collecting via surveys a list of representations typically associated with the prototypical person working in STEM. Third, the lack of diversity in the participants’ belongingness to STEM could be also mentioned as a limitation. Future studies should target more people representing those students and workers from the different STEM fields. Fourth, the different life experiences of people working and studying in STEM fields could be also noted as a constraint when comparing both types of participants’ opinions on the topic. Upcoming research could overcome this limitation by asking through closed-ending statements more concrete topics to participants with different life trajectories.

Breaking down stereotypes about people working in STEM is crucial. These stereotypical portrayals tend to discourage many girls, but also some boys to enroll in several STEM fields. Future research on this topic should therefore continue investigating how young people already in STEM perceive the typical person working in STEM occupations. Furthermore, a survey could be designed in order to delve further into the way young people already in STEM perceive the different STEM fields. In addition, these portrayals could inspire young people (particularly girls) and encourage them to enter the field.

### Ideas for Intervention

There is a lack of recognition of women’s contributions to STEM fields and a dearth of female role models making outstanding contributions. It is therefore necessary for teachers and didactic materials to raise awareness of women’s contributions to the STEM field across the different stages of the educational system. In addition, afterschool curricular STEM activities could be designed to increase both boys’ and girls’ interest in STEM fields by making them more accessible and familiar (Koch and Gorges, 2016). Inviting STEM professionals into schools to talk to students about the day-to-day work they carry could be a good strategy for this purpose. Moreover, more interventions aimed at changing how mass media reproduce the stereotypical portrayal of the ideal person working in STEM are required.

## Ethics Statement

At the time of realization of fieldwork, ethical approval was not required as per local legislation. In addition, participants were not exposed to any physical, psychological, professional or otherwise risk, as they only were posed questions regarding their vision on certain aspects of their personal experience.

All procedures performed in it studies were in accordance with the ethical standards of the institutional and/or national research committee and with the 1964 Helsinki declaration and its later amendments or comparable ethical standards. Informed written consent was obtained from all individual participants included in the study.

## Author Contributions

MS conceptualized, designed, and coordinated the realization of the study and wrote the first draft of the manuscript. SF wrote most of the method section and make insightful comments with regards the justification of the study. JM-C, MR-d-Z, MJR, and LA contributed to the development of the results section. All authors contributed to the development of fieldwork and data analysis and manuscript revision, read, and approved the submitted version.

## Conflict of Interest Statement

The authors declare that the research was conducted in the absence of any commercial or financial relationships that could be construed as a potential conflict of interest.
